# Epigenetics, Heredity, and Aging

**DOI:** 10.1093/geroni/igaa057.2619

**Published:** 2020-12-16

**Authors:** Eric Greer

**Affiliations:** Boston Children’s Hospital/Harvard Medical School, Boston, Massachusetts, United States

## Abstract

Longevity has long been shown to be regulated by genetic and environmental factors. We recently showed that longevity in the nematode Caenorhabditis elegans can also be regulated by the transmission of epigenetic information. We have shown that several chromatin modifying enzymes have a transgenerational non-Mendellian effect in worms on the longevity of their descendants. I will discuss some of our recent work attempting to decipher how epigenetic modifications can regulate complex phenotypes, including longevity, and how this non-genetic information can be transmitted across generations.

